# A multilevel intervention to promote colorectal cancer screening among community health center patients: results of a pilot study

**DOI:** 10.1186/1471-2296-10-37

**Published:** 2009-05-29

**Authors:** Karen E Lasser, Jennifer Murillo, Elizabeth Medlin, Sandra Lisboa, Lisa Valley-Shah, Robert H Fletcher, Karen M Emmons, John Z Ayanian

**Affiliations:** 1 Section of General Internal Medicine, Boston Medical Center and Boston University School of Medicine, Boston, MA, USA; 2 Department of Medicine, Cambridge Health Alliance and Harvard Medical School, Cambridge, MA, USA; 3 Centers for Disease Control and Prevention, Atlanta, GA, USA; 4 Department of Community Affairs, Cambridge Health Alliance and Harvard Medical School, Cambridge, MA, USA; 5 Department of Gastroenterology, Cambridge Health Alliance and Harvard Medical School, Cambridge, MA, USA; 6 Department of Ambulatory Care and Prevention/Harvard Medical School and Harvard Pilgrim Health Care, Boston, MA, USA; 7 Dana Farber Cancer Institute/Harvard School of Public Health, Boston, MA, USA; 8 Division of General Medicine, Brigham and Women's Hospital and Department of Health Care Policy, Harvard Medical School, Boston, MA, USA

## Abstract

**Background:**

Colorectal cancer screening rates are low among poor and disadvantaged patients. Patient navigation has been shown to increase breast and cervical cancer screening rates, but few studies have looked at the potential of patient navigation to increase colorectal cancer screening rates.

**Methods:**

The objective was to determine the feasibility and effectiveness of a patient navigator-based intervention to increase colorectal cancer screening rates in community health centers. Patients at the intervention health center who had not been screened for colorectal cancer and were designated as "appropriate for outreach" by their primary care providers received a letter from their provider about the need to be screened and a brochure about colorectal cancer screening. Patient navigators then called patients to discuss screening and to assist patients in obtaining screening. Patients at a demographically similar control health center received usual care.

**Results:**

Thirty-one percent of intervention patients were screened at six months, versus nine percent of control patients (p < .001).

**Conclusion:**

A patient navigator-based intervention, in combination with a letter from the patient's primary care provider, was associated with an increased rate of colorectal cancer screening at one health center as compared to a demographically similar control health center. Our study adds to an emerging literature supporting the use of patient navigators to increase colorectal cancer screening in diverse populations served by urban health centers.

## Background

Colorectal cancer is the second leading cause of cancer death in the United States (US). In 2008, an estimated 148,810 people will be diagnosed with colorectal cancer, and it is estimated that 49,960 will die of the disease. [1] Current guidelines from the U.S. Preventive Services Task Force [2] recommend screening individuals age 50 until age 75 with one of the following tests: flexible sigmoidoscopy every 5 years, colonoscopy every 10 years, or fecal occult blood test (FOBT) every year. Despite the availability of these effective screening tests [3-7] a large proportion of Americans are still not being screened. [8-10] Patients at greatest risk of not being screened include racial and ethnic minorities,[10,11] patients with Medicaid or no health insurance,[8,12,13] those who are foreign born,[12,14] and patients with low socioeconomic status [15] – groups that are commonly served by community health centers.[8,16]

In a prior qualitative study of community health centers at Cambridge Health Alliance that included patients from Brazil, Portugal, the Azores, Cape Verde and Haiti, [17] large immigrant groups in Massachusetts and elsewhere in the US, we found that the following factors prevented patients from being screened for colorectal cancer: 1) lack of trust in doctors; 2) lack of symptoms; 3) lack of a doctor's recommendation for screening and 4) fatalistic views about cancer. Few physicians were aware that lack of trust and fatalistic beliefs about cancer were barriers to screening for their patients. Physicians typically cited comorbid medical conditions and numerous psychosocial stressors as the main reasons why patients did not receive colorectal cancer screening.

We used these findings to inform the development of a patient navigator-based intervention. Patient navigators are people selected from the community who are trained to guide patients through the health care system to receive appropriate services.[18] A type of care management, patient navigation encompasses a wide range of advocacy and coordination activities.[19] Most published research on patient navigators has focused on breast and cervical cancer screening, showing that navigation increases the rate of patient completion of screening and follow-up evaluation.[20,21]

Several studies, all conducted in New York City, have shown that patient navigation can increase rates of colorectal cancer screening among urban minority patients.[18,21-24] Our study adds to the existing literature by including Haitian Creole and Portuguese-speaking patients, and patients in a geographic area other than New York. We report the results of a pilot study to assess the feasibility of using patient navigators to increase rates of colorectal cancer screening among community health center patients in Massachusetts.

## Methods

### Study setting and sample

Cambridge Health Alliance (CHA) is a Primary Care Practice-Based Research Network (PBRN)[25] including 15 community health centers. The health centers predominantly serve a multi-cultural, low-income population in Cambridge, Somerville, and Everett, MA. We selected one health center to pilot-test the intervention, and a demographically similar health center to serve as the control health center. The CHA institutional review board approved the study protocol. The institutional review board provided a waiver of informed consent, since the study was promoting an established screening standard and primary care providers (PCPs) were able to identify patients who were not appropriate to contact.

Using an electronic clinical data system (Meditech), we identified patients aged 52–80 who appeared to be unscreened for colorectal cancer. We included patients age 75–80 because at the time of the study, age 80 was considered to be the upper age limit of screening by the U.S. Preventive Services Task Force. We chose to begin at age 52 instead of age 50 (the age at which guidelines suggest that screening begin), because we sought consistency with the Healthcare Effectiveness Data and Information Set (HEDIS) measure on colorectal cancer screening. [26,27] The unscreened patient report used in our study also served as the basis for our ambulatory quality improvement colorectal cancer screening measure. We based eligibility for colorectal cancer screening on a modified version of the most recent HEDIS measure. US health plans utilize HEDIS measures to assess performance on important dimensions of care, including cancer screening. We modified the denominator of the measure to include any patient aged 52–80 who had one visit to a primary care physician in a community health center in each of the two previous years. The numerator included any patient who received colonoscopy in the past 10 years, sigmoidoscopy or barium enema in the past five years, or fecal occult blood testing (FOBT) during the prior year. Using this definition, 47% of eligible patients in our network of community health centers received colorectal cancer screening in the year 2006. Since the data report did not capture tests performed outside of Cambridge Health Alliance, or FOBT cards that were not billed, we suspect that the true screening rate was higher than 47%.

We limited our intervention group to patients who spoke English, Portuguese, Spanish or Haitian Creole and who received care at one center in Somerville, MA. We excluded patients of two primary care providers (PCPs) at the intervention center: one PCP who was a study investigator (KEL), and one PCP who was leaving the health center at the time of the study. The control group consisted of a random sample of similarly defined patients (speaking the same languages and unscreened for colorectal cancer based on the abovementioned definition) at another health center in Somerville.

### Study Procedures

Because the electronic data system did not capture diagnostic tests performed outside of the health center network, one investigator (KEL) reviewed the medical records of all patients at both the intervention and control health centers who appeared unscreened in the data report to confirm that they were, in fact, unscreened. After reviewing 196 medical records at the intervention center and 191 medical records at the intervention center, we identified 93 intervention patients and 90 control patients who had not received colorectal cancer screening according to the criteria specified above.

We asked each of the eight PCPs at the intervention center to review their list of unscreened patients and to identify any patient who they deemed inappropriate for telephone outreach, based on the following criteria: 1) patient has a medical contraindication to screening or a short life expectancy so that they do not warrant screening[25] 2) the patient will be out of the country continuously for at least three months during the period of navigation 3) the patient had severe cognitive or mental impairment, and no one who can be identified as a caretaker or proxy and 4) other reason as designated by the PCP.

Of the 93 unscreened patients, PCPs deemed 38 (41%) to be inappropriate for outreach for the following reasons: patient has a long history of refusing screening (n = 16), patient with medical comorbidity (n = 7), gastrointestinal symptoms or gastrointestinal workup in progress (n = 6), mental illness or substance abuse (n = 5), other reasons (n = 4; patient uninsured, out of the country, or moving). Fourteen of the 38 patients deemed ineligible for outreach were uninsured.

### Intervention

The remaining 55 patients were eligible to receive the intervention. We sent letters by first-class mail, signed by each PCP, notifying patients that they were overdue for colorectal cancer screening, and that a patient navigator would be calling them. The mailing also included a colorectal cancer screening brochure designed by the Harvard Center for Cancer Prevention and the Massachusetts Colorectal Cancer Working Group ("Take Control: Get Tested for Colorectal Cancer"). The brochure, written at a sixth-grade reading level, offered patient-oriented information about the reasons for screening, the different screening modalities, and lifestyle changes to lower risk of colorectal cancer. We sent brochures to patients in English, Portuguese, Spanish, or French (for Haitian Creole-speaking patients).

The study patients were also eligible to receive navigation from navigators speaking English and Spanish, Portuguese, and Haitian Creole, respectively. The navigators were based in the hospital's Department of Community Affairs; they did not have a presence at the intervention health center. The navigator who worked with English and Spanish-speaking patients was originally from Nicaragua, had completed college, and had extensive experience doing community health outreach. She was also a trained certified nurse's assistant (CNA). The Portuguese-speaking navigator had been a masters-level clinical psychologist in Brazil, and was an experienced community health worker. The Haitian navigator was also an experienced community health worker, and worked as a medical assistant in a local community health center. All of the navigators were women, and were age 47, 42, and 37, respectively.

The navigators attended a two day training program in October 2007. The training program included lectures and interactive role plays about the following subjects: 1) the principles of motivational interviewing [28] 2) colorectal cancer and how patients can be screened for it; 3) logistics ("how-to," pros, and cons) of FOBT cards and colonoscopy 4) prevention of colorectal cancer (including prevention by removal of adenomas) 5) use of open vs. closed questions, reflective listening, and summarizing; 6) assessment of patient's readiness for screening and 7) approaches for patients who refuse screening (pre-contemplation), are willing to think about it (contemplation), or are ready to act (action).[28] We chose to frame the intervention around a "stages of change" model as other cancer prevention studies have successfully employed this model.[29]

During the study implementation, the project manager (who also attended the training sessions) audited between one and five patient calls by each navigator for adherence to a calling script and for motivational interviewing techniques. The patient navigators and the project manager also met on a weekly basis to discuss challenges arising during the outreach calls and to review the use of motivational interviewing techniques.

Over a three week period in October 2007, the patient navigators made between 8 and 11 attempts to call each patient on different days (weekdays and weekends) and at different times (morning, afternoon, and evening) until they reached a patient. The navigators also left at least two messages for the patient, either on the answering machine or with a family member.

Once the navigator reached a patient, the navigator discussed the need for colorectal cancer screening with the patient, the screening options of colonoscopy vs. FOBT cards, and the advantages and disadvantages of each test. The navigators did not discuss other screening test options, such as flexible sigmoidoscopy and barium enema, since such options were not routinely offered to patients by their PCPs.

If a patient was interested in completing FOBT cards, the navigator reviewed the FOBT instructions with the patient and mailed FOBT cards and illustrated instructions to patients by first-class mail. The navigator also offered to review the FOBT instructions with the patient over the phone as soon as the patient received the FOBT cards. If a patient did not return the FOBT cards within four weeks, the navigator called the patient to provide support and to address barriers to completion.

For patients who were interested in pursuing colonoscopy, the navigators described the test in detail and the project manager contacted the patient's PCP to arrange a colonoscopy referral. Based on the patient's comorbid medical conditions, the PCP either referred the patient directly for colonoscopy or for a routine appointment with a gastroenterologist to discuss colonoscopy. Patients with any of the following conditions were not eligible for direct referral: sleep apnea, obesity (BMI > 30), previous history of anesthesia problems, congestive heart failure, presence of an automatic implanted cardiac defibrillator, renal failure (as defined by the PCP), and warfarin use for any reason. For patients referred directly to colonoscopy, a registered nurse (LV) called the patient, educated him/her about the procedure and the bowel preparation, and mailed instructions for the bowel preparation to the patient. The patient did not require a medical visit prior to the colonoscopy procedure. The gastroenterology office placed reminder calls to all patients one day prior to their procedure. Due to medico-legal concerns, the navigators did not escort patients home after the colonoscopy. In the event that a patient did not have someone to escort them home, the navigators advised them to complete FOBT cards instead.

At the control health center, patients eligible for colorectal cancer screening received usual care. PCPs offered patients screening on an ad-hoc basis during primary care visits. Unlike the PCPs at the intervention center, the PCPs at the control center did not review their lists of unscreened patients. At both health centers, PCPs had some decision support to promote colorectal cancer screening in the Epic electronic medical record. The electronic record includes a health maintenance grid which flags age-appropriate patients who have not received colorectal cancer screening. The PCPs at the control health center could also refer patients directly for colonoscopy at the time of the study, but they did not have access to patient navigators to advise patients on screening options or to assist them in completing the test.

### Measures

The primary outcome of the study was completion of colorectal cancer screening at six months. While the intervention focused on the completion of colonoscopy or a set of three FOBT cards from home, patients who completed any of the following during the study period were considered to have been screened: colonoscopy, sigmoidoscopy or barium enema, or FOBT cards. One of the investigators (KEL) conducted a non-blinded chart review to determine completion of colorectal cancer screening tests.

### Process Evaluation

During the study, the navigators maintained paper records in which they documented details of their interactions with patients, including the patients' readiness to be screened, barriers to screening, and actions that were taken to promote screening. The project manager entered these data into a Microsoft Access database.

### Statistical Methods

We included all patients at the intervention center in an intention-to-treat analysis, regardless of whether they were designated by their PCP to receive navigation, or whether a navigator successfully reached the patient. Using the χ^2 ^test, we compared screening rates at six months among intervention patients and control patients. We chose to analyze the data at six months because the wait for a screening colonoscopy at the time of the study was on the order of weeks, and we assumed that patients would have had sufficient time to complete their colonoscopy during the six-month period.

## Results

Table 1 shows the demographic characteristics of the intervention and control center patients. The patients at both centers were of similar age, race (note that race data were missing for 7 persons), and insurance status. Of those patients who had insurance, the majority at both sites had Medicaid or free care (66% at the intervention center and 51% at the control center). At the time of the study, after being determined ineligible for other payment options, Massachusetts residents were able to apply for help paying for health center bills from the Massachusetts uncompensated (free) care pool. The non-English speaking patients at both sites were mostly Portuguese speaking, with small numbers of Spanish and Haitian Creole speaking patients.

**Table 1:** Community Health Center Patient Characteristics

**Variable**	**Intervention**	**Control**	**Chi-square p-value**
	**n = 93**	**N = 90**	
Female (%)	63.4	75.6	.08
			
Mean Age (SD)	60.6 (6.6)	60.9 (7.1)	.54
			
Race (%)			
White	67.0	71.8	.50
Non-White	33.0	28.2	
			
Insurance coverage (%)			
No coverage	24.7	17.8	.25
Coverage	75.3	82.2	
			
Language used in visit (%)			
English	51.6	53.3	.82
Non-English	48.4	46.7	

### Screening Outcomes

**Figure 1:**
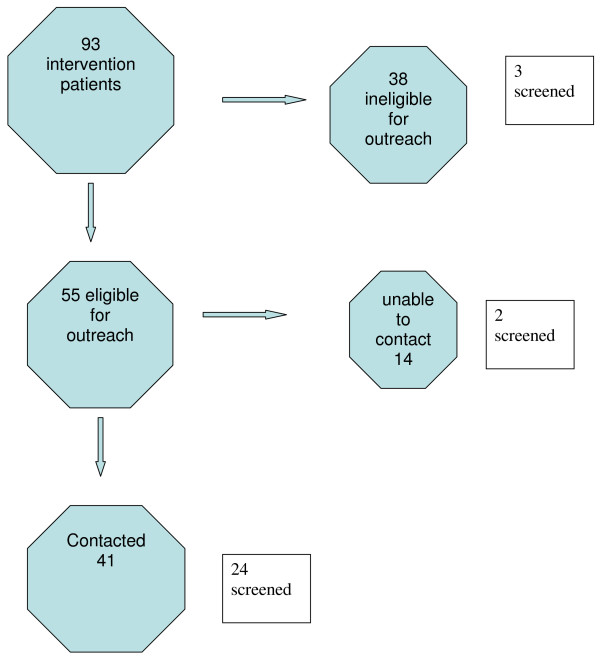
**Flow diagram of intervention patients**.

Table 2 shows the main study results. Patients in the intervention center were much more likely to be screened within six months than patients in the control group (31% vs. 9%, χ^2 ^p < .001). Due to small numbers we did not present P values for comparisons between the different types of screening (FOBT and colonoscopy). Three of the 38 patients (8%) whom PCPs at the intervention site deemed ineligible for outreach were screened at six months (Figure 1).

**Table 2:** Colorectal Cancer Screening Results

**Variable**	**Intervention**	**Control**	**Chi-square p-value**
	**n = 93**	**n = 90**	
	(%)	(%)	
			
Screened for colorectal cancer at 6 months	31.2	8.9	.0002
			
Screened by FOBT	17.2	7.8	*
Positive tests	0	3.3	*
			
Screened by colonoscopy	14.0	1.1	*
Adenomas	4.3	0	*

Of the 29 patients screened at the intervention site, 16 completed FOBT cards, and 13 completed colonoscopy. Among patients who completed FOBT cards, all tests were negative. Of those patients who completed colonoscopy, three had high-risk lesions; one patient had a tubulovillous adenoma, one patient had four tubular adenomas, and another had a 35 mm. tubular adenoma. A fourth patient had two small tubular adenomas. Among the eight patients screened at the control site, seven completed FOBT cards and one completed colonoscopy. Three patients at the control site had positive FOBT results, but only one of these patients completed a follow-up colonoscopy within six months.

### Process Outcomes

Of the 55 patients who were offered navigation, the patient navigators were unable to contact 14 (25%) after between eight and eleven attempted telephone calls. Two of these 14 patients (14%) were screened at six months, while 24 of the 41 patients (59%) whom the navigators were able to contact were screened at six months. For patients reached by the navigator, the median number of contacts was five (range 1–16). Patients received, on average, about four hours of telephone outreach. Patients who received more contact (eight or more calls) were no more likely to be screened than those who received less contact (fewer than eight calls).

In their discussions with patients, the navigators learned that many patients had not been screened because their PCP had not taken enough time to educate them about colorectal cancer screening. For example, one patient stated, "my doctor asked me if I wanted to have it (colonoscopy) done, and I said no and that was it." The patient noted that the PCP did not explore her reasons for declining screening. In addition, patients related not being able to take time off from work to undergo colonoscopy.

## Discussion

We found that a patient navigator-based intervention was associated with an increased rate of colorectal cancer screening at one health center as compared to a demographically similar control health center. Almost one-third of intervention patients were screened at six months versus nine percent of control patients. Our study adds to an emerging literature supporting the use of patient navigators to increase colorectal cancer screening in diverse populations served by urban health centers.[19,22-24]

While our intervention was effective, it did not achieve the screening rates observed in other studies. For example, studies by Chen et al[18] and Christie et al[24] found that over 50% of navigated patients completed colonoscopy. These studies offered patient navigation only after a patient had been referred for screening colonoscopy by their PCP, which may explain their higher screening rates. In addition, these studies excluded patients who required a gastrointestinal clinic visit for pre-screening evaluation. Jandorf et al[22] also achieved higher screening rates, in both the intervention and control groups. It is possible that the higher screening rates observed in all of these navigation studies could partially be attributed to secular trends. In New York City, where all three of these studies were conducted, 1.25 million people were screened in 2007, up from 826,000 in 2003, with the biggest rates of increase in minority communities.[30]

Our study was limited by the fact that only 41 (44%) of 93 unscreened patients at the intervention health center were actually contacted by a patient navigator. The PCPs at the intervention site identified 38 patients (41%) as inappropriate for outreach. While some of the PCPs reasons for excluding patients were legitimate, such as medical comorbidity, gastrointestinal symptoms or gastrointestinal workup in progress, and mental illness or substance abuse (our navigators were not trained to deal with these special populations), some of the patients who were excluded may have been good candidates for patient navigation services. Such patients included those with a long history of refusing screening and the uninsured. By excluding these patients, we may have underestimated the potential impact of patient navigation. Our study is also limited by small sample size, which precluded us from examining the individual effects of different components of the intervention (letter versus navigation) and from performing exploratory subgroup analyses.

Unlike prior studies of patient navigation, which included mostly Hispanic and African American patients, our study included immigrants from Brazil, Portugal, the Azores, and Haiti. Our inability to contact a substantial proportion (25%) of patients, which decreased the effectiveness of the intervention, may be due to the fact that many patients travel back and forth to their country of origin. The PCPs at the intervention site were often unaware of their patients' migratory patterns, and hence did not exclude such patients from being outreached. These patients also experience housing instability.

Unmeasured differences between the two health centers could account for the differential screening rates. In addition, the PCPs at the control center did not have an opportunity to identify patients whom they deemed inappropriate for screening. We attempted to account for this difference by including all of the intervention center patients in an intent-to-treat analysis. A further potential source of bias is the fact that our qualitative study of barriers to colorectal cancer screening [17] included one PCP from the intervention site, and no PCPs from the control site. We doubt that a one-hour interview conducted with a PCP in 2005 would have significantly affected his colorectal cancer screening practices.

## Conclusion

This study supports the feasibility and effectiveness of a patient navigator intervention to increase colorectal cancer screening rates in a community health center serving ethnically and linguistically diverse patients. Future studies will need to examine the cost-effectiveness of such an intervention, and a randomized trial would confirm the effectiveness of patient navigation for immigrant groups who have not been previously studied.

## Competing interests

The authors declare that they have no competing interests.

## Authors' contributions

KL, principal investigator, led clinic recruitment, led analysis, and wrote and edited drafts of the manuscript. EM, project manager, oversaw training and supervised the patient navigators. JM and SL navigated the patients, and LV talked to patients about the open access colonoscopy procedure. RF, JA, and KE participated in the initial study design and interpretation of findings. All authors read and approved the final manuscript.

## Pre-publication history

The pre-publication history for this paper can be accessed here:


